# Are Cerebrospinal Fluid Biomarkers Useful in Predicting the Prognosis of Multiple Sclerosis Patients?

**DOI:** 10.3390/ijms12117960

**Published:** 2011-11-16

**Authors:** Alberto Gajofatto, Matilde Bongianni, Gianluigi Zanusso, Maria Donata Benedetti, Salvatore Monaco

**Affiliations:** 1 Section of Clinical Neurology, Department of Neurological, Neuropsychological, Morphological and Motor Sciences, University of Verona, Verona 37129, Italy; E-Mail: mariadonata.benedetti@ospedaleuniverona.it; 2 Section of Neuropathology and The Brain Bank at the University of Verona, Department of Neurological, Neuropsychological, Morphological and Motor Sciences, University of Verona, Policlinico G.B. Rossi, Piazzale L.A. Scuro 10, Verona 37134, Italy; E-Mails: matilde.bongianni@univr.it (M.B.); gianluigi.zanusso@univr.it (G.Z.)

**Keywords:** multiple sclerosis, biomarkers, cystatin C, 14-3-3 protein, tau protein

## Abstract

Multiple sclerosis (MS) is the prototypical inflammatory demyelinating disorder of the central nervous system (CNS). Although many advances have been made in the comprehension of its pathogenesis, the etiology is still unknown. The complexity of MS reflects in the extreme variability of the clinical manifestations and clinical course both between and within patients, in addition to immunopathological mechanisms and response to treatment. Several prognostic factors have been suggested in large scale studies, but predictions in individual cases are difficult to make. Cerebrospinal fluid (CSF) biomarkers, such as 14-3-3, tau, and cystatin C are promising sources of prognostic information with a good potential of quantitative measure, sensitivity, and reliability. However, none has shown sufficient reproducibility to be applied in clinical practice. Here we review the current literature addressing the above mentioned biomarkers as MS severity predictors at an early stage.

## 1. Introduction

Multiple sclerosis (MS) is the most common chronic demyelinating disorder of the central nervous system (CNS). Pathogenesis is characterized by a complex interplay between genetic, autoimmune and environmental factors, but etiology is unknown. The biological complexity of MS is paralleled by the extreme variability of the clinical picture. Although 10–20% of patients have a progressive disease from onset (primary progressive MS), the typical clinical course is relapsing-remitting. In these patients the initial event of acute or subacute neurological disturbance is indicated as clinically isolated syndrome (CIS), although not all CIS cases necessarily convert to MS. Indeed, the natural history of CIS and early MS is highly variable and unpredictable at the individual level. When patients are diagnosed with a CIS, clinicians have to deal with many questions and challenges regarding differential diagnosis, the likelihood of conversion to MS, and treatment decisions. Typical findings on brain magnetic resonance imaging (MRI), oligoclonal bands in the cerebrospinal fluid (CSF), and/or evoked potentials suggestive of demyelination are associated with an increased risk of conversion to MS. Even in patients with early MS, many questions remain open regarding the likelihood of future relapses and disability. In some cases disease-modifying treatments might prevent the development of MS and disability, but in others they could be superfluous [1].

Studies of the clinical features and natural history of CIS and early MS use different groups of patients, either from observational investigations or from short-term therapeutic reports. In a natural history study of a group of CIS patients in London, UK, the percentage of subjects who developed clinically definite MS (CDMS) was 43% at 5 years, 59% at 10 years, and 68% at 14 years [2]. Notably, the presenting symptoms did not seem to affect the rate of conversion to definite MS. A study of 308 patients from the Gothenburg, Sweden, database found that 45 out of 220 patients with a CIS still had a CIS at the 25-year follow-up [3]. Patients with efferent lesions had twice the risk of a later diagnosis of MS than those without efferent lesions, and the likelihood of being wheelchair-bound was 2.8 times higher with efferent than non-efferent lesions, and 1.9 times higher with incomplete than complete remission of the CIS. Male gender, incomplete remission and high relapse rate in the first 5 years of MS were predictive of subsequent severe disability. Another natural history study of 1215 patients with MS in Lyon, France, found a longer period to the second episode with optic neuritis presentation than with either brainstem or spinal cord presentations [4]. Slow progression to disability milestones also occurred in patients with optic neuritis at presentation, in those with good recovery from the CIS, in those with a long period before the second relapse, and in those with few relapses in the first 5 years. Overall, however, the CIS characteristics had little effect on delay to the second episode and little, if any, effect on subsequent progression.

The presence and number of lesions on brain MRI has an effect on the course of a CIS, with a higher risk of clinically definite MS in patients with an abnormal scan [5,6]. To a lesser extent, the number/location of baseline lesions and the rate of new lesions accrual predict the likelihood of developing subsequent disability in MS cases [7,8]. There is evidence that evoked potentials have a prognostic value in MS. Patients with a high degree of evoked potentials abnormalities have an increased risk of disability progression in the subsequent follow-up period [9].

Studies of the immune activation in patients with CIS provide important information about immunological mechanisms. An abnormal B-cell response (shown by oligoclonal IgG in the CSF) is detected in about two-thirds of patients with CIS and increases the risk of MS [10]. There is evidence that CSF oligoclonal IgM predicts a more aggressive disease course in relapsing–remitting MS [11]. Detailed studies in patients with CIS suggest there may be systemic activation of myelin-reactive T cells, which secrete proinflammatory Th-1 cytokines. Interestingly, this activation has been associated with inflammatory activity on gadolinium-enhanced MRI [12].

Besides immunological markers, several CSF proteins have been studied as potential predictors of CIS/MS disease course. Among the multitude of biomarkers which have been tested, neurofilaments seem to stand out for potential prognostic value. Current evidence suggests that neurofilament heavy and light chain proteins (NfH and NfL, respectively) concentrations are increased in the CSF of MS patients compared to age-matched normal controls, although they do not reach the levels observed in neurodegenerative and more disruptive CNS disorders. Furthermore, CSF NfH and NfL levels seem to correlate with MS clinical course (higher levels in RR MS compared to CIS and in progressive compared to RR MS) and with disease activity (higher levels during relapses compared to remission phase). Finally, it has been shown that CSF NfH and NfL concentrations may correlate with disease severity both at the time of lumbar puncture and at subsequent follow-up time points [13–15]. Conversely, the presence of 14-3-3 protein has been associated with a higher risk of MS conversion and subsequent disability in patients with CIS [16]. Some studies have observed a correlation of CSF tau protein with disability in MS patients [17]. Recently, cystatin C has been proposed as a potential diagnostic and/or prognostic marker of CIS and MS [18–21].

## 2. Relevance of Cerebrospinal Fluid Biomarkers

Research on patients with CIS/early MS may provide new insights into early pathological changes and pathogenetic mechanisms that might affect the course of the disorder. In the absence of robust clinical and paraclinical variables predicting the disease course in the individual MS patient, biomarkers are promising source of information with a good potential of quantitative measure, sensitivity, and reliability. Although lumbar puncture is a relatively invasive procedure, CSF is the ideal biologic sample to analyze in CNS disorders such as MS. Considering the complex pathogenesis of MS, no single CSF molecule is expected to have prognostic significance. However, families of biomarkers representative of specific pathogenetic pathways—particularly those related to axonal/neuronal damage—may correlate with irreversible neurological dysfunction and be used as prognostic indicators to identify patients at risk of a more aggressive disease course. Furthermore, such biomarkers may help in deciding treatment initiation and monitoring therapeutic efficacy.

According to their biological role, CSF molecules of potential prognostic significance for MS may be classified as follows:

- markers of immune activation (e.g., cytokines, chemokines, antibodies, complement factors, adhesion molecules, *etc*.)
- markers of blood-brain barrier disruption (e.g., matrix metalloproteinases)
- markers of demyelination (e.g., myelin basic protein, proteolytic enzymes, *etc*.)
- markers of axonal/neuronal damage (e.g., neurofilaments, tau protein)
- markers of non-specific biological pathways (e.g., protein 14-3-3, cystatin C, *etc*.)

There is a striking amount of published data in the field of MS biomarkers and an omnicomprehensive revision of the literature on the topic is beyond the scope of the present review [22–24]. In consideration of the recent developments, the possible prognostic relevance, and the research experience of our group, here we will focus on a panel of CSF biomarkers including tau protein, 14-3-3 protein and cystatin C.

## 3. 14-3-3 Protein

14-3-3 protein belongs to a family of highly homologous acidic polypeptides of low *M_r_* (30 kDa) encoded by distinct genes. There are seven known mammalian 14-3-3 isoforms (β, γ, ɛ, η ζ, σ, and θ) mainly existing as homo- or heterodimeric complexes, which coordinate adaptation in protein-protein interactions, act as activators or suppressors, and regulate the subcellular localization of proteins. Though ubiquitous, 14-3-3s are abundant in the human brain, where they represent about 1% of total cytosolic proteins [25,26]. These proteins have attracted interest because they are involved in important cellular processes such as signal transduction, cell division, growth, adhesion, differentiation, apoptosis, stress response and malignant transformation. In the human brain, 14-3-3s may function primarily in cell-signaling pathways or as second-messenger modifiers in neurons. Owing to their abundant expression, damage to CNS tissue may potentially cause leakage of 14-3-3 proteins into the CSF following cellular disruption. It is largely established that the ɛ and γ 14-3-3 isotypes are highly expressed in the CSF of patients with Creutzfeldt-Jacob disease (CJD), and that their detection has considerable diagnostic and prognostic value [27,28]. The ɛ and γ isoforms are also increased in the CSF of subjects with neurological disorders characterized by active axonal degeneration, but not in inflammatory and demyelinating CNS disorders. Notably, in a number of neurodegenerative disorders, increased CNS expression and/or tissue deposition of 14-3-3 proteins occur in the absence of concurrent CSF release. In Alzheimer’s disease (AD), the γ and ɛ isotypes are overexpressed in several brain regions, 14-3-3ζ is bound to neurofibrillary tangles, and 14-3-3η is a component of amyloid plaques [29]. However, 14-3-3s are essentially negative in the CSF of AD, with the exception of one study reporting a positive 14-3-3η essay in a small number of patients [30]. In neurodegenerative disorders with sequestration and aggregation of α-synuclein, such as Parkinson’s disease and dementia with Lewy bodies, 14-3-3 γ, ɛ, ζ, and θ isoforms are complexed with α-synuclein, but not expressed in the CSF [31,32]. CSF 14-3-3s have been investigated in several inflammatory/demyelinating disorders, with a wide range of results.

In 1999 Satoh *et al.* reported the presence of 14-3-3 protein in the CSF of a patient affected by relapsing-remitting MS in an active stage and suggested that 14-3-3 protein might be expressed in neurons and glial cells and released into the CSF during acute stages of the disease [33]. Subsequently, Irani and Kerr observed a significant correlation between CSF 14-3-3 expression and neurological disability in a small group of patients with acute transverse myelitis, a condition sharing relevant pathogenetic features with MS, being even part of the clinical spectrum of the disorder [34].

Martínez-Yélamos *et al.* reported that a positive CSF 14-3-3 assay at the first neurological event suggestive of CIS, might be a reliable, although low-sensitive, indicator of conversion to clinically definite MS and severe neurological disability [13,35].

Colucci *et al.* suggested that the detection of 14-3-3s in the CSF could be a predictor of the severity of the disease and also a useful marker to identify MS patients at high risk of developing severe disability [36]. Bartosik-Psujek and co-workers found that the presence of 14-3-3 and increased tau protein levels in the CSF of MS patients positively correlated with the IgG index and this association was present in both relapsing remitting MS (RRMS) and progressive MS patients [37]. These findings favor the hypothesis that inflammation may induce 14-3-3 and tau proteins release in the CSF of MS patients. On the contrary, our group found a positive 14-3-3 assay coupled with normal tau protein level in the CSF of most MS/CIS cases, including patients with inactive disease and with a long time-interval from acute attack to CSF collection. Presence of 14-3-3 did not correlate with clinical outcomes and was independent of CSF oligoclonal bands and IgG index status. In addition, 14-3-3 protein isoforms expressed in MS/CIS patients (predominantly β and ζ) are substantially different from isoforms expressed in patients with CJD and ALS (predominantly γ and ɛ), which are characterized by prominent neuronal death [20,38]. This observation together with the 14-3-3/tau dissociation in the CSF (a pattern characterized by presence of 14-3-3 protein and normal tau levels) challenge the hypothesis that 14-3-3 is a marker of neuronal/axonal loss in MS. To date, the association between 14-3-3 and poor clinical outcome is not established, due to conflicting results across studies, and, on the other hand, CSF 14-3-3 expression is not constantly correlated to IgG index increase and oligoclonal bands positivity, thus suggesting that 14-3-3 may be not a marker of humoral immunity activation.

*In vitro* studies have shown that 14-3-3 protein interacts with vimentin and glial fibrillary acidic protein in cultured human reactive astrocytes derived from MS demyelinating lesions, and that astrocytes and oligodendrocytes, at the site of demyelinating lesions, show increased immunoreactivity for 14-3-3, suggesting that these cells might be the source of 14-3-3 in the CSF of MS patients [39,40]. Taken together, the 14-3-3 assay coupled with the characterization of 14-3-3 isotypes in the CSF of MS patients may provide valuable information on ongoing pathological processes, including demyelination, astrocytosis and neurodegeneration, thus providing support to therapeutic options.

## 4. Tau Protein

Tau is a microtubule-binding phosphoprotein, which plays an important role in axonal locomotion and intraneuronal transport. Tau proteins include six isoforms, with *Mr* ranging 45 to 65 kDa, differing for the presence of either three or four repeats in the carboxy-terminal region of the molecule and the presence of one or two inserts in the amino-terminal domain [41].

In normal conditions, the CSF expresses low levels of tau protein (up to 250–500 pg/mL), while tau concentration is elevated in neurological disorders characterized by ongoing neuronal and axonal degeneration, such as CJD, stroke, and encephalitis. In AD and other tauopathies, hyperphosphorylated tau accumulates within the neurofibrillary tangles, and is released in the CSF, representing a valuable diagnostic and prognostic indicator of cognitive dysfunction [42–44].

Contrasting results have appeared in the literature with regard to the significance of CSF tau in MS. Kapaki *et al.* reported increased CSF tau concentrations in 36 patients with MS compared with 29 non-matched controls (controls were 28 years older than MS patients). Increased CSF tau concentration was mainly driven by the 11 patients with secondary progressive and the 10 patients with primary progressive subtypes of MS, while the levels found in the 15 patients with relapsing-remitting subtype were similar to controls [45].

Another study found increased CSF tau levels in MS as compared to controls. The authors concluded that the increase of tau protein may reflect axonal injury in MS patients [46]. Martinez-Yelamos and coworkers described a group of early relapsing-remitting MS patients in which increased CSF tau was predictive of poor short-term outcome [14]. However, these findings are in contrast with those reported by Jimenez-Jimenez *et al.* and by Fiorini *et al.* who did not find significant differences between the CSF tau levels of controls and patients with MS [20,47]. These data suggest that tau protein concentration in the CSF is frequently normal in unselected MS cases, and that increased tau expression may be a marker of primary and secondary progressive forms of the disorder.

## 5. Cystatin C

Cystatin C is a protease inhibitor highly expressed by choroidal and leptomeningeal cells and localized in glial and neuronal cells. Cystatin C counteracts the action of cathepsins, a family of lysosomal proteins released by activated microglia/macrophages, and it is thought to have a major role in modulating immune cell activation and inflammation-driven cell death [48]. Cystatin C has been found to be downregulated in the CSF of a number of inflammatory conditions affecting the central nervous system and the peripheral nervous system [18,49].

There is increasing evidence of a possible role of cystatin C in the pathogenesis of MS. CSF levels of cystatin C may mirror the extent of inflammation in early disease stages, a factor which is known to influence the extent of later neurodegeneration and axonal loss. In an animal model of CNS demyelination, Ma *et al.* showed an increased expression of cathepsin B and cystatin C in white matter astrocytes and microglia [50]. These authors suggested that during active stages of demyelination, microglia and macrophages produce cathepsins while astrocytes release their powerful inhibitor, cystatin C. After myelin disruption, myelin debris must be cleared by activated microglia and macrophages before the naked axons can be effectively remyelinated. The dominance of cathepsins over cystatin C levels may result in disregulation of cathepsins activity at later stages of demyelination, causing impairment of remyelination [50]. Decreased levels of cystatin C, coupled with increased activity of cathepsin B, were found by Nagai and coworkers in the CSF of MS patients on relapse [18]. In contrast, our group reported a significant increase of CSF cystatin C concentration in patients with CIS and MS compared to controls [20]. Recently, we explored the possible significance of CSF cystatin C in a study of the prognostic factors in 53 patients with first-episode acute myelitis (AM). Information regarding demographics, clinical status, laboratory work-up, spine and brain MRI, and electrophysiological assessment was collected, and tau, 14-3-3 protein and cystatin C levels were assessed de novo in stored CSF samples. The prognostic value of all variables was analyzed for the following outcomes: recovery from initial event, symptoms recurrence, conversion to MS, and disability level at last follow-up visit (median follow-up of 6.2 years). Six patients remained monophasic, 5 developed recurrent myelitis, and 42 (79%) converted to MS. Sensory level absence, no sphincter involvement, abnormal brain MRI, spinal cord lesions shorter than 3 vertebral segments, and abnormal somatosensory evoked potentials predicted MS conversion. Forty-seven percent of patients with pyramidal dysfunction at onset and 40% of patients with relapses during follow-up had significant disability at last visit compared to 10% of patients without pyramidal manifestations and none of the patients without exacerbations. Tau and 14-3-3 proteins were not significantly associated with any of the outcomes, while in the subgroup of patients with exacerbations there was a significant correlation between CSF cystatin C levels and the degree of neurological disability at last visit [38]. The research was then extended to 39 patients with any first-episode of neurological disturbance compatible with the onset of MS, or CIS, such as optic neuritis, brainstem/cerebellar syndromes, and hemispheric syndromes, in addition to acute myelitis. At CIS presentation the spinal cord was clinically involved in 25 patients, the brain stem/cerebellum in 12, the optic nerve in 4, the cerebral hemisphere in 3, while 5 patients had a multifocal CIS. After a mean follow-up of 6.8 ± 3.4 years, 7 patients did not have further symptoms or dissemination of MRI lesions in time, whereas 32 converted to MS. Pyramidal system involvement at onset and moderate to severe neurological impairment at nadir were associated with incomplete CIS recovery. Somatosensory and motor evoked potentials suggestive of demyelination at CIS onset, and the occurrence of at least one relapse every 2 years during follow-up were associated with a higher risk of significant disability at last visit in patients who developed MS. Interestingly, CSF cystatin C concentration was significantly higher in patients who converted to MS compared to those who remained CIS. In addition, CSF cystatin C levels were correlated to the expanded disability status scale (EDSS) score at the end of follow-up in patients who initially had a CIS with spinal cord involvement [51].

## 6. Conclusions

Biomarkers are promising tools for prognostic predictions in MS. Ideally, reliable prognostic studies of biomarkers should fulfill some key methodological requirements, particularly: (1) prospective design; (2) sufficient follow-up; (3) adequate biomarker and outcome measurement; (4) clinical significance of the biomarker (*i.e.*, good correlation and consistency with relevant clinical outcomes); (5) reproducibility. Currently, no biomarker study has simultaneously shown such characteristics.

In the present review, we summarize the potential diagnostic and prognostic significance of three CSF proteins in patients with CIS/early MS: (1) 14-3-3 protein, showing upregulation of distinct isoforms, which deserve further investigation to elucidate a possible prognostic significance; (2) tau protein, which is helpful in distinguishing demyelinating disorders from diseases characterized by prominent axonal loss; (3) cystatin C, a marker changing its CSF expression in accordance with disease stage and progression. In proposing this panel of biomarkers, we are further comforted by the long follow-up, over time periods of up to 10 years, long after the disease presentation. In addition, our strategy to explore CSF samples, collected well after the acute episodes, is at variance with most studies, which explore biomarkers during acute relapses and, therefore, are characterized by a prevalence of inflammatory proteins.

Among explored candidates, cystatin C may have a major role in the pathogenesis of MS, and could be used as a potential source of prognostic information. In particular, high cystatin C concentrations in the CSF may identify MS patients with an unfavorable clinical course of the disease. Further data in this area of research may provide new insights into the pathogenesis of irreversible neurological disability in MS.

## References

[1] Miller D., Barkhof F., Montalban X., Thompson A., Filippi M. (2005). Clinically isolated syndromes suggestive of multiple sclerosis, part I: Natural history, pathogenesis, diagnosis, and prognosis. Lancet Neurol.

[2] Brex P.A., Ciccarelli O., O’Riordan J.I., Sailer M., Thompson A.J., Miller D.H. (2002). A longitudinal study of abnormalities on MRI and disability from multiple sclerosis. N. Engl. J. Med.

[3] Eriksson M., Andersen O., Runmarker B. (2003). Long-term follow up of patients with clinically isolated syndromes, relapsing-remitting and secondary progressive multiple sclerosis. Mult. Scler.

[4] Confavreux C., Vukusic S., Adeleine P. (2003). Early clinical predictors and progression of irreversible disability in multiple sclerosis: An amnesic process. Brain.

[5] Morrissey S.P., Miller D.H., Kendall B.E., Kingsley D.P., Kelly M.A., Francis D.A., MacManus D.G., McDonald W.I. (1993). The significance of brain magnetic resonance imaging abnormalities at presentation with clinically isolated syndromes suggestive of multiple sclerosis. A 5-year follow-up study. Brain.

[6] Jacobs L.D., Kaba S.E., Miller C.M., Priore R.L., Brownscheidle C.M. (1997). Correlation of clinical, magnetic resonance imaging and cerebrospinal fluid findings in optic neuritis. Ann. Neurol.

[7] Minneboo A., Barkhof F., Polman C.H., Uitdehaag B.M., Knol D.L., Castelijns J.A. (2004). Infratentorial lesions predict long-term disability in patients with initial findings suggestive of multiple sclerosis. Arch. Neurol.

[8] Li D.K., Held U., Petkau J., Daumer M., Barkhof F., Fazekas F., Frank J.A., Kappos L., Miller D.H., Simon J.H. (2006). MRI T2 lesion burden in multiple sclerosis: A plateauing relationship with clinical disability. Neurology.

[9] Invernizzi P., Bertolasi L., Turatti M., Bianchi M.R., Gajofatto A., Benedetti M.D. (2011). Prognostic value of multimodal evoked potentials in multiple sclerosis: The EP score. J. Neurol.

[10] Sandberg-Wollheim M., Bynke H., Cronqvist S., Holtås S., Platz P., Ryder L.P. (1990). A long-term prospective study of optic neuritis: Evaluation of risk factors. Ann. Neurol.

[11] Sola P., Mandrioli J., Simone A.M., Ferraro D., Bedin R., Annecca R., Venneri M.G., Nichelli P.F., Merelli E. (2011). Primary progressive *versus* relapsing-onset multiple sclerosis: Presence and prognostic value of cerebrospinal fluid oligoclonal IgM. Mult. Scler.

[12] Jensen J., Langkilde A.R., Fenst C., Nicolaisen M.S., Roed H.G., Christiansen M., Sellebjerg F. (2004). CD4 T cell activation and disease activity at onset of multiple sclerosis. J. Neuroimmunol.

[13] Malmeström C., Haghighi S., Rosengren L., Andersen O., Lycke J. (2003). Neurofilament light protein and glial fibrillary acidic protein as biological markers in MS. Neurology.

[14] Salzer J., Svenningsson A., Sundström P. (2010). Neurofilament light as a prognostic marker in multiple sclerosis. Mult. Scler.

[15] Kuhle J., Leppert D., Petzold A., Regeniter A., Schindler C., Mehling M., Anthony D.C., Kappos L., Lindberg R.L.P. (2011). Neurofilament heavy chain in CSF correlates with relapses and disability in multiple sclerosis. Neurology.

[16] Martínez-Yélamos A., Saiz A., Sanchez-Valle R., Casado V., Ramón J.M., Graus F., Arbizu T. (2001). 14-3-3 protein in the CSF as prognostic marker in early multiple sclerosis. Neurology.

[17] Martínez-Yélamos A., Saiz A., Bas J., Hernandez J.J., Graus F., Arbizu T. (2004). Tau protein in cerebrospinal fluid: A possible marker of poor outcome in patients with early relapsing-remitting multiple sclerosis. Neurosci. Lett.

[18] Nagai A., Murakawa Y., Terashima M., Shimode K., Umegae N., Takeuchi H., Kobayashi S. (2000). Cystatin C and cathepsin B in CSF from patients with inflammatory neurologic diseases. Neurology.

[19] Irani D.N., Anderson C., Gundry R., Cotter R., Moore S., Kerr D.A., McArthur J.C., Sacktor N., Pardo C.A., Jones M., Calabresi P.A., Nath A. (2006). Cleavage of cystatin C in the cerebrospinal fluid of patients with multiple sclerosis. Ann. Neurol.

[20] Fiorini M., Zanusso G., Benedetti M.D., Righetti P.G., Monaco S. (2007). Cerebrospinal fluid biomarkers in clinically isolated syndromes and multiple sclerosis. Proteomics Clin. Appl.

[21] Staun-Ram E., Miller A. (2011). Cathepsins (S and B) and their inhibitor Cystatin C in immune cells: Modulation by interferon-β and role played in cell migration. J. Neuroimmunol.

[22] Bielekova B., Martin R. (2004). Development of biomarkers in multiple sclerosis. Brain.

[23] Teunissen C.E., Dijkstra C., Polman C. (2005). Biological markers in CSF and blood for axonal degeneration in multiple sclerosis. Lancet Neurol.

[24] Tumani H., Hartung H.P., Hemmer B., Teunissen C., Deisenhammer F., Giovannoni G., Zettl U.K., BioMS Study Group (2009). Cerebrospinal fluid biomarkers in multiple sclerosis. Neurobiol. Dis.

[25] Boston P.F., Jackson P., Kynoch P.A., Thompson R.J. (1982). Purification, properties, and immunohistochemical localisation of human brain 14-3-3 protein. J. Neurochem.

[26] Boston P.F., Jackson P., Thompson R.J. (1982). Human 14-3-3 protein: Radioimmunoassay, tissue distribution, and cerebrospinal fluid levels in patients with neurological disorders. J. Neurochem.

[27] Hsich G., Kenney K., Gibbs C.J., Lee K.H., Harrington M.G. (1996). The 14-3-3 brain protein in cerebrospinal fluid as a marker for transmissible spongiform encephalopathies. N. Engl. J. Med.

[28] Piubelli C., Fiorini M., Zanusso G., Milli A., Fasoli E., Monaco S., Righetti P.G. (2006). Searching for markers of Creutzfeldt-Jakob disease in cerebrospinal fluid by two-dimensional mapping. Proteomics.

[29] Layfield R., Fergusson J., Aitken A., Lowe J., Landon M., Mayer R.J. (1996). Neurofibrillary tangles of Alzheimer’s disease brains contain 14-3-3 proteins. Neurosci Lett.

[30] Wiltfang J., Otto M., Baxter H.C., Bodemer M., Steinacker P., Bahn E., Zerr I., Kornhuber J., Kretzschmar H.A., Poser S. (1999). Isoform pattern of 14-3-3 proteins in the cerebrospinal fluid of patients with Creutzfeldt-Jakob disease. J. Neurochem.

[31] Xu J., Kao S.-Y., Lee F.J.S., Song W., Jin L.-W., Yanker B.A. (2002). Dopamine-dependent neurotoxicity of alfa-synuclein: A mechanism for selective neurodegeneration in Parkinson disease. Nat. Med.

[32] Berg D., Riess O., Bornemann A (2003). Specification of 14-3-3 *proteins* in Lewy bodies. Ann. Neurol.

[33] Satoh J., Kurohara K., Yukitake M., Kuroda K. (1999). The 14-3-3 protein detectable in the cerebrospinal fluid of patients with prion-unrelated neurological diseases is expressed constitutively in neurons and glial cells in culture. Eur. Neurol.

[34] Irani D.N., Kerr D.A. (2000). 14-3-3 protein in the cerebrospinal fluid of patients with acute transverse myelitis. Lancet.

[35] Martínez-Yélamos A., Rovira A., Sànchez-Valle R., Martínez-Yélamos S., Tintorè M., Blanco Y., Graus F., Montalban X., Arbizu T., Saiz A. (2004). CSF 14-3-3 protein assay and MRI as prognostic markers in patients with clinically isolated syndrome suggestive of MS. J. Neurol.

[36] Colucci M., Roccatagliata L., Capello E., Narciso E., Latronico N., Tabaton M., Mancardi G.L. (2004). The 14-3-3 protein in multiple sclerosis: A marker of disease severity. Mult. Scler.

[37] Bartosik-Psujek H., Archelos J.J. (2004). Tau protein and 14-3-3 are elevated in the cerebrospinal fluid of patients with multiple sclerosis and correlate with intrathecal synthesis of IgG. J. Neurol.

[38] Gajofatto A., Monaco S., Fiorini M., Zanusso G., Vedovello M., Rossi F., Turatti M., Benedetti M.D. (2010). Assessment of outcome predictors in first-episode acute myelitis: A retrospective study of 53 cases. Arch. Neurol.

[39] Satoh J., Yamamura T., Arima K. (2004). The 14-3-3 protein ɛ isoform expressed in reactive astrocytes in demyelinating lesions of multiple sclerosis binds to vimentin and glial fibrillary acidic protein in cultured human astrocytes. Am. J. Pathol.

[40] Kawamoto Y., Akiguchi I., Kovács G.G., Flicker H., Budka H. (2004). Increased 14-3-3 immunoreactivity in glial elements in patients with multiple sclerosis. Acta Neuropathol.

[41] Buée L., Bussière T., Buée-Scherrer V., Delacourte A., Hof P.R. (2000). Tau protein isoforms, phosphorylation and role in neurodegenerative disorders. Brain. Res. Brain Res. Rev.

[42] Hulstaert F., Blennow K., Ivanoiu A., Schoonderwaldt H.C., Riemenschneider M., de Deyn P.P., Bancher C., Cras P., Wiltfang J., Mehta P.D. (1999). Improved discrimination of AD patients using beta-amyloid(1-42) and tau levels in CSF. Neurology.

[43] Andreasen N., Minthon L., Clarberg A., Davidsson P., Gottfries J., Vanmechelen E., Vanderstichele H., Winblad B., Blennow K. (1999). Sensitivity, specificity, and stability of CSF-tau in AD in a community-based patient sample. Neurology.

[44] Mattsson N., Zetterberg H., Hansson O., Andreasen N., Parnetti L., Jonsson M., Herukka S.K., van der Flier W.M., Blankenstein M.A., Ewers M. (2009). CSF biomarkers and incipient Alzheimer disease in patients with mild cognitive impairment. J. Am. Med. Assoc.

[45] Kapaki E., Paraskevas G.P., Michalopoulou M., Kilidireas K. (2000). Increased cerebrospinal fluid tau protein in multiple sclerosis. Eur. Neurol.

[46] Terzi M., Birinci A., Cetinkaya E., Onar M.K. (2007). Cerebrospinal fluid total tau protein levels in patients with multiple sclerosis. Acta Neurol. Scand.

[47] Jiménez-Jiménez F.J., Zurdo J.M., Hernanz A., Medina-Acebrón S., de Bustos F., Barcenilla B., Sayed Y., Ayuso-Peralta L. (2002). Tau protein concentrations in cerebrospinal fluid of patients with multiple sclerosis. Acta Neurol. Scand.

[48] Reed C.H. (2000). Diagnostic applications of cystatin C. Br. J. Biomed. Sci.

[49] Yang Y., Liu S., Qin Z., Cui Y., Qin Y., Bai S. (2009). Alteration of cystatin C levels in cerebrospinal fluid of patients with Guillain-Barré syndrome by a proteomical approach. Mol. Biol. Rep.

[50] Ma J., Tanaka K.F., Yamada G., Ikenaka K. (2007). Induced expression of cathepsins and cystatin C in a murine model of demyelination. Neurochem. Res.

[51] Gajofatto A., Fiorini M., Monaco S., Turatti M., Bianchi M.R., Fiaschi A., Benedetti M.D. (2010). Predicting the natural course of clinically isolated syndromes: An exploratory study of cerebrospinal fluid biomarkers. Mult. Scler.

